# Optineurin coding variants in Ghanaian patients with primary open-angle glaucoma

**Published:** 2008-12-18

**Authors:** Yutao Liu, Stephen Akafo, Cecile Santiago-Turla, Claudia S. Cohen, Karen R. LaRocque-Abramson, Xuejun Qin, Leon W. Herndon, Pratap Challa, Silke Schmidt, Michael A. Hauser, R. Rand Allingham

**Affiliations:** 1 Center for Human Genetics, Duke University Medical Center, Durham, NC; 2 Department of Ophthalmology, Duke University Eye Center, Duke University Medical Center, Durham, NC; 3 Unit of Ophthalmology, Department of Surgery, University of Ghana Medical School, Accra, Ghana

## Abstract

**Purpose:**

Coding variants in the optineurin gene (*OPTN*, GLC1E) have been reported to play a role in primary open-angle glaucoma (POAG) in various populations. This study investigated the role of *OPTN* sequence variants in patients with POAG in Ghana (West Africa).

**Methods:**

This is a case-control study of unrelated Ghanaian POAG cases and non-glaucomatous controls. Ascertainment criteria for POAG included the presence of glaucomatous optic nerve neuropathy, associated visual field loss, and elevated intraocular pressure (IOP) in both eyes, all in the absence of secondary causes of glaucoma. Controls had normal optic nerves, visual fields, and IOP. All the coding exons of *OPTN* were polymerase chain reaction (PCR) amplified and sequenced in all 140 cases and 130 controls using an ABI 3730 DNA analyzer.

**Results:**

All the coding exons of *OPTN* were sequenced in 140 POAG patients and 130 controls. Several coding variants were identified including M98K, A134A, V147L, P292P, A301G, S321S, and E322K. Three coding variants (V147L, P292P, and A301G) have not been reported previously. There were no significant differences on the frequencies of all the identified variants between POAG cases and controls in this population.

**Conclusions:**

This is the first comprehensive study of *OPTN* in a single West African population. Our results suggest that coding variants in *OPTN* may not contribute to the risk for POAG in persons of West African descent.

## Introduction

Glaucoma is a group of disorders defined by a characteristic loss of retinal ganglion cells associated with optic nerve degeneration and visual field loss. It is the second leading cause of bilateral blindness worldwide [1]. Primary open-angle glaucoma (POAG, OMIM 137760) is the most common form of glaucoma in the United States and worldwide, afflicting approximately 67 million individuals in the year 2000 alone [1]. Well recognized risk factors for the development of POAG include elevated intraocular pressure (IOP), positive family history of glaucoma, refractive error, and race; African-Americans have a higher risk of developing POAG [1-3].

There is a strong hereditary component to glaucoma as first-degree relatives of affected individuals have a 7–10 fold higher risk of developing the disease than the general population [4]. Using genetic linkage analysis, at least 14 chromosomal loci for POAG (GLC1A- GLC1N) have been identified in family data sets and are listed by HUGO (Human Genome Organization, Geneva, Switzerland). Disease-associated sequence variants in three genes, myocilin (*MYOC*, GLC1A), optineurin (*OPTN*,**GLC1E), and WD repeat domain 36 (*WDR36*, GLC1G), have been described in POAG and other types of glaucoma [5-7]. Disease-associated mutations of myocilin are generally associated with a juvenile or early-adult form of POAG, which account for about 3%–5% of POAG patients.

The mechanistic role of *OPTN* in the pathogenesis of glaucoma remains unclear. It is widely expressed in both ocular and non-ocular tissues including but not limited to the heart, brain, placenta, liver, skeletal muscle, kidney, and pancreas. In ocular tissues, it is expressed in the trabecular meshwork, non-pigmented ciliary epithelium, retina, Schlemm's canal, and the aqueous humor [8]. The OPTN protein interacts with different proteins that are involved in apoptosis, inflammation, and vasoconstriction [8,9]. OPTN might play a neuroprotective role by reducing retinal ganglion cell susceptibility to apoptosis [8,9]. Overexpression of OPTN blocks cytochrome c release from the mitochondria and protects the cell from hydrogen peroxide-induced cell death [10]. It was also found that OPTN negatively regulates TNF-α induced NF-κB activation, leading to reduced apoptosis [9,11]. *OPTN* was originally identified as a dominant, normal tension glaucoma (NTG) gene in a large study involving 54 families [6]. These sequence variants (E50K, M98K, R545Q, and c691–692insAG) in *OPTN* were considered disease causing in the original study [6]. The variant, E50K, remains the most consistently found in association studies with normal tension glaucoma. It is found that *OPTN* E50K increases binding to TANK-binding kinase 1 (TBK1), which forms a complex to regulate TNF-α and its pro-apoptotic effects [12]. *OPTN* sequence variations have also been shown to be associated with POAG in Indian and Japanese populations [13,14] while playing a much less significant role in Caucasians [15-18].

African-American ancestry is estimated to increase the risk of POAG by fourfold to sixfold when compared with European ancestry [19]. African-Americans are largely descended from ancestors in West Africa. We have previously shown that POAG in Ghana has an earlier age of onset and appears to be more clinically severe than in the United States and Europe [20]. POAG patients with mutations in myocilin have a similar phenotype. In an earlier investigation, we reported that myocilin mutations cause approximately 4% of POAG in West Africa [21,22]. This report investigates the role of *OPTN* coding variants in POAG cases and controls in the same West African population from Ghana.

## Methods

### Subjects

This study adhered to the tenets of the Declaration of Helsinki. Informed consent was obtained from all participating individuals after explanation of the nature and possible consequences of the study. The research was reviewed and approved by the institutional review boards of all participating institutions including Duke University Medical Center (Durham, NC) and the Ghana Ministry of Health (Accra, Ghana).

Clinical data and DNA samples were collected from individuals identified with POAG and unaffected family members from Ghana. All POAG cases and controls were enrolled from either private clinics or the University of Ghana in Accra, the capital of this country. All patients recruited for the study were screened and ascertained by fellowship-trained glaucoma subspecialists (P.C., L.W.H., or R.R.A.). POAG subjects were unrelated and met the following three inclusion criteria: (1) intraocular pressure (IOP; by applanation tonometry) greater than 22 mmHg in both eyes without medications or greater than 19 mmHg on two or more medications; (2) glaucomatous optic neuropathy in both eyes; and (3) visual field loss consistent with optic nerve damage in at least one eye [23]. Glaucomatous optic nerve damage was defined as a vertical cup-to-disc ratio higher than 0.7 or a focal loss of the nerve fiber layer (notch), which is associated with a consistent glaucomatous visual field defect. Visual fields were performed using standard automated perimetry [1]. An open anterior chamber angle has been found in all of our POAG cases. Exclusion criteria included the presence of any secondary form of glaucoma including exfoliation syndrome or a history of ocular trauma. The criteria for unrelated control subjects were (1) no first degree relative with glaucoma; (2) intraocular pressure less than 22 mmHg tested by applanation tonometry on two occasions; (3) no evidence of glaucomatous optic neuropathy; and (4) normal visual field by automated perimetry or frequency doubling test (FDT).

### DNA analysis

Genomic DNA was extracted from peripheral blood by standard techniques (Gentra, Minneapolis, MN). Primers flanking each coding exon (exon 4−16) in *OPTN* were designed with Primer3 software [24] and were listed in Table 1. All sequencing was performed using appropriately selected primers and conditions optimized in a standard fashion. Platinum Taq DNA polymerase (Invitrogen, Carlsbad, CA) was used for all the polymerase chain reactions (PCRs). The PCRs were performed in MJ Research PTC-200 PCR machines (MJ Research, Waltham, MA) using a touchdown program (94 °C 3 min; then 94 °C 30 s, 65 °C 30 s, 72 °C 30 s for two cycles; 94 °C 30 s, 63 °C 30 s, 72 °C 30 s for two cycles; 94 °C 30 s, 61 °C 30 s, 72 °C 30 s for two cycles; 94 °C 30 s, 59 °C 30 s, 72 °C 30 s for two cycles; 94 °C 30 s, 57 °C 30 s, 72 °C 30 s for two cycles; 94 °C 30 s, 55 °C 30 s, 72 °C 30 s for 30 cycles; and 72 °C 3 min). Sequencing reactions were performed using BigDye^®^ Terminator v3.1 Cycle Sequencing Kits (Applied Biosystems, Foster City, CA) and run on the ABI 3730 DNA analyzer (Applied Biosystems). All the sequence analysis was done by using the Sequencher 4.8 (Gene Codes, Ann Arbor, MI). All suspected variations were confirmed by bidirectional sequencing. The M98K variant was subsequently genotyped using a custom TaqMan SNP (single nucleotide polymorphism) Genotyping Assay (Applied Biosystems). The E322K variant could not be genotyped by TaqMan SNP genotyping due to the lack of available assay from ABI (Applied Biosystems).

**Table 1:** List of PCR primers for *OPTN* DNA sequencing in Ghanaian POAG cases and controls.

***OPTN* exon**	**Forward primer sequence**	**Reverse primer sequence**	**PCR product size (bp)**	**Mg^2+^ concentration**
4	TAAGTATTAGCAATCGCCAA	AGTGCAAAGGGATGGCATTT	340	2.0 mM
5	CATCAGATCAAGTCCACTTT	GGAGTCTAGACACGTAAGAT	340	2.0 mM
6	ATGGTGCCCAGCCTTAGTTT	CAATCCTTGGCTTGTGTTGA	340	1.5 mM
7	CATCTGAATGTTTGGAAGCT	TATTCTGGAAAGATCCTGGT	340	1.5 mM
8	ATACTGAACAGGGCATTGTC	GTGGTTGCACAATCCTGGAA	300	1.5 mM
9	GATCCTTTATCCCAATTGTA	TTGAATTCAGTGGCTGGACT	282	2.0 mM
10	TTGATTCACCAGCCAGTCTT	GCTCACACATTAACTGGAAC	400	1.5 mM
11	TGCATTCATAAACCCTACAG	TAGGACTCCTTCAGATAAGT	400	1.5 mM
12	TTGAGAGTAAGAAATGCTAG	GATTTAGTGAAGGATTCATG	340	1.5 mM
13	ATGTTGCCCAGGCTTGTCTC	CACCATTGCTTTCCAATGCG	420	1.5 mM
14	GGATACAGCACTACCTCCTC	TCAGGAACGTCTTTGGACAG	300	1.5 mM
15	GCTCAGTGTTGTCATGTTTC	GGAATCCATTGTAGAGAATG	240	1.5 mM
16	TCGCCATCTGTTCTTCAAGT	AAAAGCACAACTCTTGGAGG	269	2.0 mM

### Statistical analysis

Sequencing data were analyzed as mentioned previously [25,26]. Briefly, allele frequencies for each coding variant in POAG cases and controls were compared by logistic regression with adjustment for age and sex (SAS software; SAS Institute Inc., Cary, NC). Analysis of the Hardy–Weinberg equilibrium (HWE) was performed separately for patients and controls using GDA (Genetic Data Analysis) software according to previously described methods [25]. SNP genotypes were coded according to a log-additive model in which the relative risk for carriers of two variant (minor) alleles when compared with the reference group (homozygous wild-type) was assumed to be the square of the relative risk for carriers of one variant. A p value less than 0.05 was considered statistically significant.

## Results

DNA and relevant clinical data were collected from 140 POAG cases and 130 controls. Detailed information about this data set has been described previously [25,26]. Briefly, the mean ages for POAG cases and controls were 63±12.6 years old and 59.4±12.6 years old, respectively. The percentages of POAG cases and controls that were female were 50% and 57%, respectively. The mean IOPs for POAG cases and controls were 33.6±11.1 mmHg and 17.2±2.8 mmHg, respectively.

Seven coding variants were identified in *OPTN* in this data set. There were three synonymous (A134A, P292P, and S321S) and four non-synonymous (M98K, V147L, A301G, and E322K) coding variants (Table 2). The M98K, A134A, S321S, and E322K variants have been reported in other populations [6,15,16,27-30]. The other three coding variants (V147L, P292P, and A301G) are novel and have not been previously reported. The M98K variant in exon 5 was detected in 43 cases (two homozygotes and 41 heterozygotes) and 33 controls (two homozygotes and 31 heterozygotes; p>0.05). One individual with POAG had a novel V147L variation in exon 6 as a heterozygote. The age-onset for this patient was 59 years old. Another POAG patient whose age-onset was relative early at the age of 40 years had a novel A301G variant in exon 10 as a heterozygote. None of the controls exhibited either of these two sequence variants. However, neither of these variants showed any statistically significant difference in allele frequencies between cases and controls (p>0.05; Table 3). Seven POAG patients and one control had the heterozygous E322K variant in exon 10. These seven patients have relatively high IOP (>30 mmHg). However, the allele frequency of this variant is not significantly different between POAG cases and controls (p>0.05). None of the remaining synonymous coding variants (A134A, P292P, and S321S) showed significantly different allele frequencies between POAG cases and controls. No HWE problem was detected for any of the coding variants in *OPTN* (p>0.05).

**Table 2:** *OPTN* DNA sequence variants in Ghana POAG patients.

**Location**	**Sequence change**	**Codon change**	**SNP ID**
Exon 5	c.603A>T	M98K	rs11258194
Exon 6	c.712C>A	A134A	
Exon 6	c.749G>C	V147L	
Exon 9	c.1186G>A	P292P	
Exon 10	c.1212C>G	A301G	
Exon 10	c.1273C>T	S321S	
Exon 10	c.1274G>A	E322K	rs523747

Nucleotides are numbered as in GenBank AF420371.

**Table 3:** Allele frequencies of sequencing variants in *OPTN* in POAG patients and control subjects.

**Marker**	**Allele**	**Allele Frequency**		**Sample size**		p*
		**Control**	**POAG**	**Control**	**POAG**	
M98K	A	0.859	0.831	124	133	>0.05
	T	0.141	0.169			
A134A	C	0.977	0.968	130	140	>0.05
	A	0.023	0.032			
V147L	G	1.000	0.996	130	140	>0.05
	C	0	0.004			
A301G	C	1.000	0.996	130	136	>0.05
	G	0	0.004			
S321S	C	0.987	0.993	120	136	>0.05
	T	0.013	0.007			
E322K	G	0.996	0.975	130	140	0.07
	A	0.004	0.025			

The asterisk denotes that the p value is from logistic regression with the adjustment for age and sex. POAG: primary open-angle glaucoma.

## Discussion

In this study, we report the contribution of *OPTN* variants in POAG cases in Ghana. We found a total of seven *OPTN* coding variants, four of which were non-synonymous and three were synonymous coding variants. Of these variants, three have not been previously reported. The large number of novel *OPTN* variants is likely due to two factors. First, our study is the first comprehensive study to examine the sequencing variants of *OPTN* in a single western African population. Second, African populations may have greater genetic diversity compared with the non-African populations reported to date [31,32]. The African populations arise from ancient and recent population expansion and contraction, short and long range migrations, and population admixture.

The genetic association between *OPTN* variants and glaucoma has been extensively studied in different populations. Mutations in *OPTN* account for about 16% of familial POAG with normal tension in the original study [33]. These sequence variants (M98K, E50K, R545Q, and c691–692insAG) were considered disease-causing originally [6]. E50K has been consistently confirmed by many studies as disease-causing [8,9,33]. R545Q has not been confirmed in other populations [9,33]. Other variants have also been reported in POAG patients including T34T, E163E, 553–5C, and E322K [9,28,34].

Coding variant, E322K (rs523747), was found to be present in more POAG cases than controls, although it failed to show any statistical significance. Interestingly, this SNP has been shown to have a minor allele (A) frequency of 0.05, which is unique and found only in sub-Saharan African HapMap samples. It is not polymorphic in either Caucasian or Asian (Chinese or Japanese) HapMap samples. Ayala-Lugo and colleagues [28] also identified this variant in samples of African ancestry including African American, Ghanaian, Nigerian, and the Caribbean. They did not find any significant difference in allele frequency between cases of open-angle glaucoma and controls with the sample size of 81 patients and 88 controls. Our study has a much larger data set of 140 POAG cases and 130 controls, all from Accra, Ghana. All the patients with E322K variants have relative high IOP (>30 mmHg) and the age-onset ranges from 43 to 85 years old. Both Ayala-Lugo’s and our studies suggest that E322K variant in *OPTN* may not contribute to the increased risk of POAG in the population of African ancestry.

The M98K *OPTN* variant is the most common variant reported in most studies [13,33]. Similar to others, we have found that the M98K variant is the most prevalent in this population. The M98K variant was originally described as a risk-associated alteration for hereditary adult-onset POAG [6]. This variant has been reported to be a potential risk factor for NTG or POAG in some Asian populations [6,14,15,27,34-36]. However, most studies have not found an association between this variant and either NTG or POAG [16-18,37-42]. Consistent with these studies, we also failed to identify an association of the M98K variant with POAG in the Ghanaian population.

Both V147L and A301G variants are novel. Each of them was found in a single POAG case and none of the controls. The age-onset for both patients is relatively early (40 and 59 years old). The IOP for these two patients is also relatively high, more than 30 mmHg. Although not statistically significant, it remains possible that these rare variants might contribute to the pathogenesis of POAG. Additional studies in larger data sets will be necessary to corroborate the role of these *OPTN* variants in glaucoma. Although we did not find any significant association between *OPTN* coding variants and Ghanaian POAG patients with elevated IOP, it is important to check these variants, especially the novel ones, in other populations with a large data set and to check their functional impact with the OPTN protein.

In conclusion, we have identified seven genetic variants of *OPTN* of which three are novel in the Ghanaian population. Two non-synonymous coding variants were found only in POAG patients and not in controls. The study presented here is the first large scale comprehensive analysis of *OPTN* variants in the Ghanaian (West African) POAG patients with elevated intraocular pressure.
